# The Endoscopic Management of Pain in Chronic Pancreatitis

**DOI:** 10.1155/2012/860879

**Published:** 2012-03-05

**Authors:** Adam J. Goodman, Frank G. Gress

**Affiliations:** Division of Gastroenterology and Hepatology, Department of Medicine, SUNY Downstate Medical Center, Brooklyn, NY 11203, USA

## Abstract

Pain resulting from chronic pancreatitis is often debilitating and difficult to manage. Many approaches have been used to treat these patients, including narcotic analgesia, antidepressants, pancreatic enzymes, octreotide, denervation procedures, such as celiac plexus block, and various palliative, decompression, or drainage procedures. Many of these procedures can be performed endoscopically, while others require a more invasive, surgical approach. The effectiveness of these therapies is not only highly variable but also often controversial. This review will discuss the endoscopic options for pain management in patients with chronic pancreatitis and their utility in treating this difficult disease.

## 1. Introduction

Pain resulting from chronic pancreatitis is often difficult to manage. Many approaches have been used to treat these patients, including narcotic analgesia, antidepressants, pancreatic enzymes, octreotide, denervation procedures (most commonly CPB), and various palliative, decompression, or drainage procedures [1–13]. The effectiveness of these therapies is not only highly variable but also often controversial. Opioid analgesics are probably used most often and can treat pain effectively, but they are associated with numerous side effects, including constipation, delirium, nausea, and the potential for addiction in patients with chronic pancreatitis [14, 15]. Nonpharmacologic methods of pain control may improve quality of life and minimize drug-related side effects [14]. Endoscopic management of pain in chronic pancreatitis consists of procedures aimed at reducing neurogenic sensation, such as celiac plexus block, or drainage procedures aimed at alleviating outflow obstruction of the pancreatic duct.

## 2. Celiac Plexus Block

The celiac plexus lies anterior to the aorta at the level of the celiac artery. Most of the sensory nerves returning from the pancreas and other intraabdominal viscera pass through the celiac ganglion and splanchnic nerves. Interruption of these fibers may lessen pain in patients with chronic pancreatitis [16]. Celiac plexus block (CPB), a temporizing treatment, most commonly refers to injection of a steroid and long-acting local anesthetic into the celiac plexus to control pain associated with chronic pancreatitis. In contrast, celiac plexus neurolysis (CPN) generally refers to injection of alcohol or phenol, a more permanent agent, into the celiac axis area [16]. This technique induces a chemical splanchnicectomy that ablates the nerve fibers that transmit pain and is used in patients with pancreatic cancer; however, it is not usually employed in patients with pain mediated by chronic pancreatitis.

CPB has traditionally been performed via various percutaneous and surgical approaches [17]. Recently, the EUS-guided approach has gained acceptance since it offers the most direct access to the celiac plexus. Wiersema and coworkers [14, 16, 18] recognized the anatomic advantage that EUS provides in visualizing the celiac region and were successful in performing transgastric EUS-guided celiac plexus blocks with results similar to the more traditional approaches. The timing of the block relative to pain onset may predict response. One study which aimed to look at CPN showed that it was more effective when the block was performed early after pain onset [19]. This result was postulated to be related to contribution of visceral and somatic nerves late in the disease and pain apparently deriving mainly from the celiac plexus early on; however, it is unclear if this translates into patients with chronic pancreatitis and the use of CBP for pain relief [16, 19]. More recently, it has been proposed that direct injection into the celiac ganglia, multiple injections in the area of the ganglia, or bilateral injections around the celiac ganglia are safe and may be more beneficial in providing sustained pain relief [20–22]. These studies are contradictory, however, and better prospective trials are needed to determine if these approaches make an improvement over the standard technique of EUS-guided CPB.

Several studies have shown that EUS-guided CPB has a beneficial role in the treatment of pain induced by chronic pancreatitis [23, 24]. An initial study of 18 patients with chronic pancreatitis showed a reduction in pain in 50% (5 of 10) of EUS-guided CPB compared with 25% (2 of 8) of CT-guided blocks [23]. This improvement in pain persisted for up to 24 weeks in 30% of responders. A cost comparison showed a $200 saving for EUS-guided CPB compared with CT-guided CPB. Another report of 90 patients by the same investigators found a significant improvement in overall pain scores in 55% at 4 weeks and 8 weeks of follow-up [24]. However, a persistent benefit beyond 24 weeks was observed in only 10% of patients. Pain relief was more likely in older patients (>45 years old) and patients who had not had previous surgery for chronic pancreatitis. A recent meta-analysis aimed to look at the efficacy of CPB for improving pain in patients with chronic pancreatitis showed that the overall percentage who obtained pain relief with this procedure was 32.7% (Table 1) and that very few good quality studies exist [25]. A major issue with all of these studies is the lack of long-term follow-up. Further, prospective studies with long-term follow-up are needed to clarify what role EUS-guided CBP will play in the management of painful chronic pancreatitis.

CPB is a generally effective, safe, and well-tolerated procedure. The three most common complications are transient hypotension (20% to 40%), transient diarrhea (4% to 38%), and transient increase in pain (9%), which are expected in CPB performed via any route [16, 26, 27]. Interruption of the plexus can result in a sympathetic blockade [28]. Clinical manifestations of sympathetic blockade can include diarrhea and hypotension resulting from a relative unopposed visceral parasympathetic activity. Mesenteric vasodilation accounts for the hypotension, which resolves in approximately 2 days. Diarrhea and increase in baseline pain are also usually limited to 2 days. Less common complications include unilateral paresis or paraplegia, pneumothorax, loss of sphincter function, retroperitoneal bleeding, renal puncture, and prolonged gastroparesis [14, 16, 27, 29]. In addition, cephalic spread of the neurolytic agent may result in involvement of the cardiac nerves and plexus affecting the heart and surrounding thoracic structures [30]. Compared to alternative approaches, EUS guidance may decrease the incidence of complications because the needle does not traverse the paraspinal region or somatic nerves or traverse the diaphragm and pleural space [1, 14, 16]. Infectious complications are uncommon but potentially serious. In a series of 90 patients, only 1 patient developed an infectious complication (peripancreatic abscess), which resolved with a 2-week course of antibiotics [24]. The authors reasoned that there might have been a predisposition to infection owing to gastroduodenal colonization with bacteria because the patient was taking a proton pump inhibitor. They suggested that prophylactic antibiotics should be considered in patients who are receiving acid suppression.

## 3. ERCP-Guided Therapies

Patients with chronic pancreatitis associated with dilation of the main pancreatic duct, stone disease, or strictures may develop symptoms of severe abdominal pain. In addition to celiac plexus block, this pain can be treated endoscopically with procedures aimed at draining the main pancreatic duct, removing stones, and dilating strictures. ERCP with pancreatic sphincterotomy, dilation of strictures, placement of stents, and stone extraction has become a mainstay of therapy in patients with painful chronic pancreatitis as recent studies have shown that on average, over 65% of patients with strictures or stones treated with endoscopic therapy have shown improvement in their pain [33]. Many studies over the last 20 years have attempted to address the question as to whether endoscopic therapy for the control of pain in chronic pancreatitis is effective. The results of selected published studies on pain relief after endotherapy for chronic pancreatitis are summarized in Table 2.

Experienced practitioners with advanced ERCP skills should only perform endoscopic therapies for chronic pancreatitis. In experienced hands, endoscopic pancreatic sphincterotomy is safe and effective and allows the therapeutic endoscopist access to the main pancreatic duct. This is usually performed under direct visualization with a pull-type sphincterotome after deep cannulation and guidewire insertion. The major risks of the procedure include pancreatitis, bleeding, and perforation. In addition, there is a risk of pancreatic sphincter stenosis that is considered a late complication after pancreatic sphincterotomy [34]. Once access to the main pancreatic duct is achieved, small stones can be removed endoscopically with success [35]. However, large, impacted stones usually require extracorporeal shock wave lithotripsy (ESWL) prior to attempted endoscopic removal. ESWL is a low-risk procedure where calcific pancreatic duct stones are usually identified by X-ray prior to the procedure. Then a fluid cushion is applied to the front and back of the patient, and shock waves are passed through the identified stones. This results in fragmentation of stones in chronic calcific pancreatitis allowing the endoscopist to then attempt to obtain complete clearance of the main pancreatic duct [35]. Multiple sessions of ESWL may be required in attempt to clear the pancreatic duct, and the success rate for complete clearance of the main pancreatic duct and resolution of pain has been at best approximately 75% [36]. There are some studies to suggest that ESWL alone, without combined endoscopic therapy, may be enough in the treatment of chronic calcific pancreatitis. A randomized controlled trial by Dumonceau and colleagues in 2007 aimed to answer this question [37]. There were 55 patients in the study, and the follow-up was 2 years. They were able to show that ESWL is a safe and effective treatment alone, without combined endoscopic therapy. Most studies looking at the role of ESWL performed to date have shown mixed efficacy due to the small sample sizes in the studies. A recent meta-analysis performed by Guda et al. aimed to overcome this problem and included 17 studies from 1989 to 2002 with a total of 588 subjects. Their data showed that ESWL is both effective in clearance of stones from the pancreatic duct and in relief of pain from chronic pancreatitis [38]. In addition, there is newer literature to suggest that the timing of ERCP after ESWL may increase the success rate of this procedure [39]. This study suggests waiting at least 2 days after ESWL before attempting ERCP and ductal clearance possibly due to ESWL-induced edema of the pancreatic duct. Long-term success of these procedures is variable, and many patients will have recurrent pain attacks after short-term successful clearance of the main pancreatic duct. This is thought, at least partially, to be due to stone migration or recurrence and responds to repeated attempts at endoscopic clearance of the main pancreatic duct [40]. Other studies have suggested that endotherapy with ESWL and pancreatic duct drainage will provide long-term pain relief for up to two-thirds of patients [41]. The authors' practice is to perform ESWL on patients with chronic, calcific pancreatitis, associated with large pancreatic duct stones that are not amenable to endoscopic removal at the time of initial ERCP. After ESWL is completed, ERCP and repeat attempt at ductal decompression are then performed.

 Pancreatic duct strictures are usually caused by fibrosis around the main pancreatic duct as a result of the chronic inflammation seen in the disease process. Strictures that are focal and located towards the head and neck region of the pancreas are more amenable to endoscopic therapy. A recent prospective study was able to show a decrease in pain after ERCP with dilation and stent placement in 89% of patients; however after 2 years of follow-up 30% of patients had relapsed and required further therapy [42]. In attempt to perform more definitive therapy for pancreatic duct strictures, some investigators have attempted placing multiple stents into the main pancreatic duct in order to dilate refractory strictures and improve PD drainage. Costamagna and colleagues were able to show that during a mean follow-up of 38 months, 84% of patients remained asymptomatic. Only 5.5% of patients had a persistent stricture after multiple stenting, and only 10.5% of patients had symptomatic stricture recurrence [43].

## 4. Surgical Treatment Options

It is the prevailing belief by most endoscopists, including the authors, that endoscopic therapy should be attempted prior to surgical intervention. Endoscopic therapy is less invasive and shows similar results in the short term compared with surgical alternatives. Díte and colleagues presented data on the first randomized controlled trial comparing endoscopic versus surgical therapy, the latter consisting of resection and drainage procedures [49]. A total of 72 patients were randomized, and the initial success rate was similar in both groups. However, long-term follow-up favored complete absence of pain in the surgical group. This study was limited by the fact that ESWL was not performed, and repeat endoscopic therapy was not allowed for pain recurrence. A more recent randomized controlled trial comparing surgical drainage (pancreaticojejunostomy) versus endoscopic therapy showed that the surgical alternative was superior in improving pain in patients with chronic pancreatitis and a dilated pancreatic duct [51]. Although this study has been criticized for its small numbers, possible selection bias, and lack of rigorous endoscopic therapy, Cahen and colleagues were able to show complete or partial pain relief in 75% of surgically treated patients versus a 32% success rate in endoscopically treated patients. In addition, these investigators have recently published their 5-year follow-up results showing that 68% of the endoscopically treated patients required additional drainage procedures, compared with 5% in the surgery group (*P* = 0.001) [52]. They also report that 47% of the patients in the endoscopy group eventually received surgery. In the long term, despite comparable levels of quality of life and pancreatic function between the surgical and endoscopic management group, surgery was still superior, in terms of pain relief (80% versus 38%, *P* = 0.042). Although endoscopic therapy is still considered first-line treatment, these randomized trials comparing surgical and endoscopic therapy should give the endoscopist pause to think about the procedures being offered to a patient for the treatment of pain in chronic pancreatitis.

 In conclusion, endoscopic therapy aimed at treating pain in patients with chronic pancreatitis consists of EUS-guided celiac plexus block and therapeutic ERCP procedures combined with ESWL that are all aimed at draining an obstructed pancreatic duct. As a result they have the potential to work well in patients with large duct disease, but they do not work well in patients with small duct chronic pancreatitis. Endoscopic management is safe and effective for many patients, but pain relief is often short lived requiring multiple repeat procedures. Endoscopic technologies as well as therapeutic techniques continue to evolve, and as such, improvements will likely be seen in the endoscopic management of chronic pancreatitis in the future.

## Figures and Tables

**Table 1:** Meta-analysis of EUS-guided celiac plexus block for chronic pancreatitis. The lower end of the confidence interval was used as the overall percentage of pain relief (adapted from Kaufman et al. [25]).

Study	Pain relief reported out of total patient (average)	95% CI
Gress et al. [23]	5/10 (50%)	(0.2836–1)
Gress et al. [24]	50/90 (56%)	(0.4689–1)
Levy et al. [20]	5/13 (38%)	(0.2217–1)
O'Toole et al. [31]	20/31 (65%)	(0.4912–1)
LeBlanc et al. [21]	27/51 (53%)	(0.4215–1)
Stevens et al. [32]	16/26 (62%)	(0.3272–1)

Over all Studies	123/221 (56%)	(0.3272–1)

**Table 2:** Selected studies on pain relief with pancreatic endotherapy.

Author	Year	Number of cases	Procedure performed	Pain relief (%)
McCarthy et al. [44]	1988	33	PS, stent	80
Sauerbruch et al. [45]	1992	24	PS, stent, ESWL	50
Delhaye et al. [46]	1992	123	PS, stent, ESWL	37
Binmoeller et al. [47]	1995	93	PS, stent, ESWL	64
Adamek et al. [48]	1999	70	PS, stent, ESWL	54
Rösch et al. [33]	2002	1018	PS, stent, ESWL	85
Díte et al. [49]	2003	36	PS, stent, ESWL	65
Delhaye et al. [41]	2004	56	PS, stent, ESWL	78
Gabbrielli et al. [50]	2005	22	PS, stent, ESWL	100
Costamagna et al. [43]	2006	19	PS, stent (multiple)	84

PS; pancreatic sphincterotomy, ESWL: extracorporeal shock-wave lithotripsy.
